# Glucose, insulin, insulin receptor subunits α and β in normal and spontaneously diabetic and obese *ob/ob* and *db/db* infertile mouse testis and hypophysis

**DOI:** 10.1186/s12958-020-00583-2

**Published:** 2020-03-17

**Authors:** R.-Marc Pelletier, Hamed Layeghkhavidaki, María L. Vitale

**Affiliations:** 1 Department of Pathology and Cell Biology, Université de Montréal, Montréal, Québec Canada; 2 Department of Pathology and Cell Biology, Faculty of Medicine, Université de Montréal, Pavillon Roger Gaudry, Case Postale 6128, Succursale Centre-ville, Montréal, Québec H3C 3J7 Canada

**Keywords:** Testis, Sertoli cell, Germ cells, Type 2 diabetes, Insulin, Glucose

## Abstract

**Background:**

Type 2 diabetes touches young subjects of reproductive age in epidemic proportion. This study assesses glucose, total InsulinT, Insulin2 and insulin receptor subunits α and β in testis during mouse development then, in the spontaneously type 2 diabetes models associated with infertility *db/db* and *ob/ob* mice. IR-β and α were also assessed in spermatozoa (SPZ), anterior pituitary (AP) and serum.

**Methods:**

Serum and tissue glucose were measured with enzymatic colorimetric assays and InsulinT and Insulin2 by ELISAs in serum, interstitial tissue- (ITf) and seminiferous tubule (STf) fractions in14- > 60-day-old normal and *db/db*, *ob/ob* and wild type (WT) mice. IR subunits were assessed by immunoblotting in tissues and by immunoprecipitation followed by immunoblotting in serum.

**Results:**

*Development*: Glucose increased in serum, ITf and STf. InsulinT and Insulin2 dropped in serum; both were higher in STf than in ITf. In > 60-day-old mouse ITf, insulinT rose whereas Insulin2 decreased; InsulinT and Insulin2 rose concurrently in STf. Glucose and insulin were high in > 60-day-old ITf; in STf high insulin2 accompanied low glucose. One hundred ten kDa IR-β peaked in 28-day-old ITf and 14-day-old STf. One hundred thirty five kDa IR-α was high in ITf but decreased in STf.

Glucose escalated in *db/db* and *ob/ob* sera. Glucose doubled in ITf while being halved in STf in *db/db* mice. Glucose significantly dropped in *db/db* and *ob/ob* mice spermatozoa. InsulinT and Insulin2 rose significantly in the serum, ITf and STf in *db/db* and *ob/ob* mice. One hundred ten kDa IR-β and 135 kDa IR-α decreased in *db/db* and *ob/ob* ITf. Only 110 kDa IR-β dropped in *db/db* and *ob/ob* STf and AP. One hundred ten kDa IR-β fell in *db/db* and *ob/ob* SPZ. One hundred ten kDa *s*IR-α rose in the *db/db* and *ob/ob* mouse sera.

**Conclusion:**

Insulin regulates glucose in tubules not in the interstitium. The mouse interstitium contains InsulinT and Insulin2 whereas tubules contain Insulin2. Decreased 110 kDa IR-β and 135 kDa IR-α in the *db/db* and *ob/ob* interstitial tissue suggest a loss of active receptor sites that could alter the testicular cell insulin binding and response to the hormone. Decreased IR-β levels were insufficient to stimulate downstream effectors in AP and tubules. IR-α shedding increased in *db/db* and *ob/ob* mice.

## Background

The incidence of diabetes in adults nears 10, and 90% of the subjects are of the insulin-dependent type 2, the seventh-leading recognised cause of death in the world [1, 2]. Type 2 diabetes used to be diagnosed primarily in older subjects. Nowadays, type 2 diabetes, impaired glucose tolerance and obesity touch adolescents and young subjects of reproductive age in epidemic proportion [1]. Around 25% of type 2 diabetes men exhibit hypogonadotropic hypogonadism [3]. Anterior pituitary hormone secretion is severely perturbed in diabetic and obese subjects [4–7]. Metabolic disorders impact on the general population by altering reproduction in both sexes. Yet, the type 2 diabetes-induced impact on spermatogenesis has so far received little attention.

Elevated blood glucose is a pathological feature of diabetes mellitus associated with an inadequate control of the sugar by insulin. Because glucose is controlled by insulin in different ways in cells of the body, we aim to assess the elements of the glucose metabolism that influence the signalling pathways activated by insulin receptors in the two cellular compartments within the testis because each exerts distinct functions. For this reason, glucose, InsulinT (Total), and Insulin2 concentrations and the insulin receptor (IR) beta IR-β and alpha IR-α subunits protein content were assessed in the serum, the interstitial tissue- and seminiferous tubule-enriched fractions during the normal mouse postnatal development. Next, we investigated glucose control mechanisms peculiar to the interstitium and tubules in the leptin-deficient (*ob/ob*) [8] and leptin receptor-deficient (*db/db*) [9] mice, two spontaneously diabetic and obese infertile type 2 diabetes mouse models.

The leptin-deficient (*ob/ob*) or (*Lep*^*ob*^*/Lep*^*ob*^) mouse model develops hyperphagia, obesity, transient hyperglycaemia, high serum insulin, elevated numbers of pancreatic beta cells, lowered LH [6], GH [4] in the serum but higher hypophyseal Prl protein content [10]. Conversely, the leptin receptor-deficient *db/db* mouse model exhibits hyperleptinemia, obesity, hyperglycaemia, high insulin and decreased Prl in the serum [7] and elevated hypophyseal Prl [10]. Humans with mutations of leptin or its receptor present the phenotype of obesity and infertility [11].

Besides adipocytes, Leydig cells in humans and rodent [12] and human spermatozoa express leptin [13]. In humans, the serum leptin concentration is inversely proportional to androgen levels [14]. The *db/db* and *ob/ob* mice exhibit low testosterone levels [15]. The leptin’s inhibiting action on food intake has been ascribed to fat stores and obesity [8]. Thirty to 40% of infertility cases are said to arise from obesity [16] and an increase in body weight in the male was shown to impact the fertility in the couple [17].

Glucose uptake has been shown to drop [18] and protein synthesis [19] and oxygen uptake ceased to be stimulated by glucose in testes in which germ cells were absent either naturally during development or in response to assaults [20]. These studies revealed that roughly a third of the energy produced aerobically in the testis is directly contributed by glucose, the major source of energy supplied by spermatocytes and spermatids which metabolise the sugar through the Embden-Meyerh of pathway of glycolysis, acetyl CoA formation, and the citric acid cycle [21]. The remaining of the energy produced is supplied by endogenous substrates like lipids [22]. The lactate production is associated chiefly with the interstitial and Sertoli cells in which glucose is oxidized to CO_2_ in small amounts but predominantly through the pentose cycle and pyruvate carboxylation pathways necessary for maintaining the citric acid cycle [22].

The report of a negative correlation between glucose and human sperm motility [23] evidences an impact of the sugar on the gamete’s metabolism which albeit varies amongst species. For instance, in the mouse, glucose is required for hyper activated motility at the end of capacitation for successful fertilisation by epididymal spermatozoa [24], beyond capacitation [25] and on the sperm-oocyte fusion [26]. As well, glucose optimises capacitation and fertilization in human sperm [27]. Incubating sperm with glucose increases in vitro fertilization rates in human [28]. Sperm motility is altered in subjects with insulin-dependent diabetes [29]. The spermatozoon’s plasma membrane and the acrosome are targets for insulin [30]. Insulin has been located in Leydig cells and spermatids in rat [31] and in the human spermatozoon’s subacrosomal space, midpiece and tail [32]. Not only human ejaculated spermatozoa express the mRNA and insulin protein but, in addition, insulin is secreted by the gametes through an autocrine feedback affecting its own secretion [32]. The nuclear and mitochondrial DNA fragmentation and apoptosis in spermatozoa were higher in diabetic than in normal subjects [33] indicating detrimental effects on the germ cells development within the testis. Moreover, sperm DNA damage was said to decline embryo quality and implantation rates [34].

A single copy of insulin-coding gene was reported in the human and Guinea pig genome [35] by contrast to mouse and rat in which insulin genes are part of a two-gene system [36, 37]. The *ins2* is an ortholog to the insulin genes in other mammals including humans; *ins1* which results from a duplication of the ancestral *ins2* gene is a rodent-Murinae-specific retrogene involved in the glucose metabolic pathways [38]. In the pancreas, *ins1* and *ins2* are transcribed and both encode proinsulin peptides which are made up of signal peptide, B chain, C-peptide, and A chain. The report that only the *ins1* gene hastens the onset of type 1 diabetes in the knockout nonobese diabetic (NOD) male mice indicates that *ins1* and *ins2* gene hold different functions [39, 40]. The fact that in the above reports, the male *ins1*-carrying NOD mice were principally affected denotes a unique impact of the insulin genes in the male. We took advantage of the *db/db* and *ob/ob* mouse models to identify the individual impact of *ins1* and *ins2* in the glucose metabolic pathways within the interstitial tissue and seminiferous tubules.

Insulin is an anabolic peptide hormone of a disulfide-linked 21-amino-acid chain A and a 30-amino-acid B chain secreted by the pancreatic β cells and on which depends glucose homeostasis. The hormone’s impact on the glucose metabolism varies with the target cells but its initial action takes place through binding as a monomer to a glycoprotein receptor endowed with insulin-stimulated tyrosine kinase activity located within the target cells’ plasma membrane [41]. The insulin receptor (IR) belongs to a subfamily of receptor tyrosine kinases that encompasses the insulin-like growth factor (IGF)-I receptor and the insulin receptor-related receptor (IRR) [42]. In somatic cells, the insulin receptor is a heterotetrameric complex made up of two disulfide bond-linked extracellular α-subunits each one linked by another disulfide bond to a transmembranous β-subunit that comprises the cytoplasmic tyrosine kinase domain and the phosphorylation sites [43]. The tyrosine kinase activity of the β-subunit is constitutively inhibited by the α-subunit [42]. The insulin binding to the α-subunit releases the kinase activity in the β-subunit from the inhibition causing the transphosphorylation and conformational changes that activates signalling cascades [42].

This study shows that during development, glucose concentration augmented in the serum, the interstitium and seminiferous tubules in which glucose levels became less than in the interstitial tissue in adulthood. Total (T) Insulin and Insulin2 concentrations dropped in serum whereas in the interstitium, InsulinT rose while Insulin2 decreased in adulthood. The results show that insulin is not glucose-regulated in the interstitium in contrast to the tubules where insulin regulates glucose levels and high Insulin2 coexists with stable glucose concentrations in normal adult mouse. The *db* and *ob* mutations produced different effects on the content and fragmentation of the IR proteins and on insulin signalling. The *db* and *ob* mutation-induced downregulation of α- and β subunits decreased IR in the interstitium. Conversely, in tubules and the anterior pituitary, the α subunit was not affected but the reduced β subunits to be activated by insulin would not suffice to stimulate downstream effectors.

## Materials and methods

### Chemicals

Phenylmethane-sulfonyl fluoride (PMSF), leupeptin, aprotinin, and Lumi-lightPlus chemiluminescence detection kit were purchased from Roche (Laval, QC, Canada). Potassium bisperoxo (1, 10- phenanthroline) oxovanadate (V) [bpV (phen)] was obtained from Calbiochem (San Diego, CA, USA). Protein G agarose was from Expedeon (San Diego, CA, USA).

### Antibodies

Mouse monoclonal IgG anti-IR-β subunit from Millipore (Etobicoke, ON, Canada). Rabbit polyclonal IgG anti-IR-α subunit from Biorbyt (Cambridge, UK). Mouse monoclonal anti-IR-α subunit was from ThermoFisher, Rockford, IL, USA). HRP-conjugated anti-mouse IgG and HRP-conjugated anti-rabbit IgG were obtained from Jackson Immunoresearch Laboratories (West Grove, PA, USA).

### Animals

The mouse model provides invaluable opportunities to explore consequences of altering the coding of specific genes on precise tissue functions. However, the small size of mouse testis allowed obtaining interstitial tissue- and seminiferous tubule-enriched fractions in small amounts. Mice were first anaesthetized (urethane, 1 g/kg IP, Sigma, St-Louis, MO, USA) before decapitation next, testes and epididymides were harvested. Blood was collected, allowed to clot; serum was obtained by centrifugation at 1500 rpm (GS-6R Beckman Centrifuge, JH-3.8 Rotor) 20 min and stored at -80C. Animal use protocol was approved by University of Montreal Animal Care Committee (Protocol number 12–126).

### Normal mouse

Studies on development were carried out on the same male mice of BALB/cJ background that we used and described in our earlier study [15]. Five animals were used per age group.

### Diabetic and obese mice

These studies were carried out on the same mice as the ones we used and described in our earlier study [15]. Twenty male mice aged of 10 weeks with the leptin receptor (B6.BKS(D)-*Leprdb*/J homozygote (*db*/*db*) Stock Number 00697) mutation, 25 male mice aged of 10 weeks with the leptin (B6.Cg-*Lepob*/J homozygote (*ob*/*ob*) Stock Number 00632) mutation both experimental group on the C57BL/6 J genetic background and ten wild type (wt) mice were used to identify the consequences of diabetes and obesity resulting from distinct mutations of specific genes on selected testicular functions. Mice were purchased from Jackson Lab (Bar Harbor, ME, USA). They were housed at RT with food and water ad libitum and exposed to a 12 h: 12 h light-dark cycle.

### Isolation of seminiferous tubule-enriched fractions

Different anatomical and functional characteristics set apart the interstitium and the seminiferous tubules in the testis. Yet, in most studies, assays are performed on whole testis extracts rather than on interstitium- and/or seminiferous tubule-enriched fractions as was done in the present study. We showed that exposure to enzymes significantly alters the detection of the phosphorylated and glycosylated proteins forms within tissue samples [44]. For this reason, the interstitial tissue-enriched and seminiferous tubule-enriched fractions were obtained without a beforehand enzymatic digestion using the technical approach detailed elsewhere [15, 44]. Briefly, seminiferous tubules were mechanically teased apart from the interstitium with Dumont fine tweezers from freshly decapsulated testes in cold phosphate buffered saline (PBS: 137 mM NaCl, 3 mM KCl, 8 mM Na_2_HPO_4_, 1.5 mM KH_2_PO_4_, pH 7.4) containing 2 mM PMSF, 1 mM EGTA, 2 μg/ml leupeptin, 2 μg/ml aprotinin, 4 mM Na_3_VO_4_, 80 mM NaF and 20 mM Na4P_2_O_7_ with 10 μM bpV (phen). The resulting seminiferous tubule-interstitium solution was centrifuged 15 min at 400 rpm, (GS-6R Beckman Centrifuge, JH-3.8 Rotor) at 4C after having been allowed to decant. The interstitial tissue- (ITf) and seminiferous tubule-enriched (STf) fractions were centrifuged 10 min at 1000 rpm (GS-6R Beckman Centrifuge, JH-3.8 Rotor) at 4C. The enriched fractions were characterised under the light microscope [15, 45].

### Isolation of epididymal spermatozoa

The isolation of epididymal spermatozoa was carried out as described before [46]. Briefly, epididymides from *db/db*, *ob/ob* and WT mice were diced in cold PBS with proteases and phosphatase inhibitors, filtered through a 74 mm mesh, and centrifuged at 2000 rpm for 15 min in a GS-6R Beckman centrifuge (JH-3.8 Rotor) at 4C to recover spermatozoa. Gametes were resuspended in10 mM Tris-HCl, pH 8, containing 1 mM EDTA for 5 min to lyse epithelial and blood cells [47], washed twice, and diluted 1:1 in cold PBS with proteases and phosphatase inhibitors. Cells were sonicated in a Fisher Sonic Dismembrator (model 300; Fisher, Farmington, NY) during three 30 s intervals.

### Protein quantification

Proteins in samples were assayed using materials from BioRad (BioRad, Mississauga, ON, Canada).

### Electrophoresis and western blot analyses

Twenty to thirty μg total homogenate of sample were loaded on polyacrylamide gels, separated by 10% SDS-PAGE, transferred onto nitrocellulose membranes and subjected to western blotting as previously described [48]. In all western blot experiments, the membranes were first stained with Ponceau red to ensure equal loading. Next, membranes were blocked 1 h at 37C with 5% skimmed milk in TRIS-buffered saline (TBS: 137 mM NaCl, 27 mM KCl, 25 mM Tris-HCl pH 7.4) next, incubated with the different antibodies. The antibody dilutions were prepared in 5% skimmed milk-TBS: polyclonal anti-IR-α (2 μg/ml), monoclonal anti-IR-α (5 μg/ml), monoclonal anti-IR-β (1.25 μg/ml). Next, membranes were washed in TBS containing 0.05% Tween 20 and incubated 1 h with a corresponding secondary antibody conjugated to HRP at RT. The antigen-antibody complexes were detected by chemiluminescence. The intensity of the immunoreactive bands was quantified by laser scanning with the public Scion Image Software (Scioncorp, MD, USA).

### Serum and tissue glucose measurements

Serum, STf and ITf glucose content was measured using an enzymatic (Mutarotase-GOD) calorimetric technique (Autokit Glucose Wako, Wako, TX, USA)) according to the manufacturer’s instructions. Tissue factions were prepared as described by Koya et al. [49] with some modifications. Briefly, STf and ITf were sonicated in 6 N perchloric acid while in an ice bath. The acid homogenates were centrifuged at 14,000 *g* and the supernatant used for glucose determination. Ten μl serum or testicular fraction homogenates were mixed with 1.5 ml colour reagent and next incubated 10 min. The absorbance of samples and standards was measured at 505 nm against the blank.

### Insulin measurements in serum and tissues

Insulin content in serum and tissue fractions was measured with commercially available ELISAs. STf and ITf were homogenized with a tissue grinder in an acid ethanol solution (180 mM HCl in 70% ethanol; 0.01 ml /mg tissue) while on ice [50]. Tissue lysates were sonicated (Fisher Sonic Dismembrator) 3 X 15 s before being centrifuged 5 min at 10,000 *g* at 4C. The supernatant was recovered for insulin determination. Total insulin (Insulin T) levels were measured with an ELISA kit from ALPCO Diagnostics (Salem NH, USA) and insulin 2 levels were measured with Ins2 ELISA kit from Aviva Systems Biology (San Diego, CA, USA).

### Immunoprecipitation

One milligram protein from serum was incubated either with 2 μl rabbit polyclonal anti-IR-α (100 μg/ml) or sample buffer (used as control) and left overnight on a rotating drum at 4C. The following day, samples were incubated with 50 μl protein-G agarose for 3 h at 4C and centrifuged 5 min at 14,000 *g* at 4C next, the supernatant was discarded. The pellets were washed with 10 mM TRIS, pH 7.4, 150 mM NaCl, 1% Triton X-100, and 1 mM EDTA and the supernatant discarded. Lastly, pellets were resuspended in 50 μl of 2 x SDS-PAGE loading buffer heated 10 min at 50C and centrifuged 2 min (14,000 *g*) at 4C. The supernatant was transferred to a new tube, boiled 5 min before western blotting. Membranes were incubated overnight at 4C with either polyclonal anti-IR-α or monoclonal anti-IR-α.

### Data and statistical analysis

The statistical analyses were done with Stata software (Stata Corporation, College Station, TX, USA). The data were evaluated with the Student’s t test or analysis of variance (ANOVA) followed by Tukey honest significant difference (HSD) test according to the number of groups to be compared.

## Results

The biochemical analyses were carried in mouse interstitial (ITf) and seminiferous tubule-enriched fractions (STf) obtained without a beforehand enzymatic digestion [15, 44] to preserve the proteins integrity and enable detection of otherwise undetectable isoforms [44] in the samples. The enriched fractions were characterised under the light microscope to ascertain the “cleanness” of the preparations and the good preservation of cellular elements [45].

### Postnatal development studies

#### Glucose

##### Serum

**Fig. 1:**
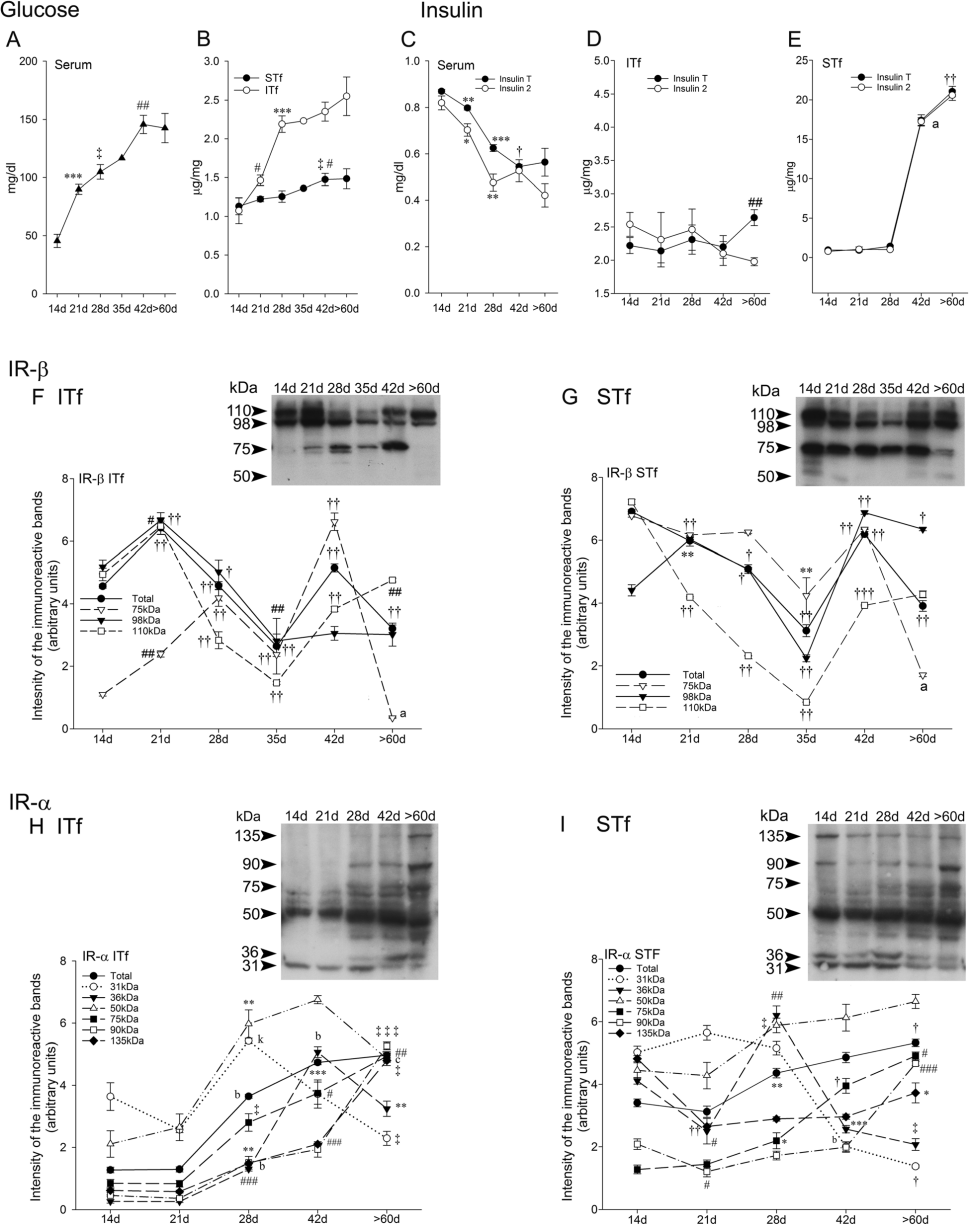
Glucose, Insulin T and Insulin 2 levels in serum, interstitial tissue- (ITf) and seminiferous tubule-enriched (STf) fractions (**a-e**) and insulin receptor subunits β and α levels in ITf and STf (**h** and **i**) during mouse postnatal development. For measurements in serum samples, three animals per age group were used. For measurements in tissue samples, five animals were used per age group. Values shown are the mean ± SEM. **a** and **b** glucose concentration expressed **a** in mg/dl in serum and **b** in μg/mg respectively in ITf and STf from 14 to > 60 days (**d**) *postpartum* in normal mice. **a** Circulating glucose concentrations significantly increased in (***P < 0.0005) 21d (**d**) versus (vs) 14d then, (‡*P* < 0.03) in 28 vs 21d and again (##*P* < 0.002) in 42 vs 28d. **b** The glucose concentration was significantly augmented in ITf (#*P* < 0.02) in 21 vs 14d (***P < 0.0005) then, in 28 vs 21d whereas in STf, the rise was significant (#*P* < 0.02) in 42 vs 14d and (‡*P* < 0.03) in 42 vs 28d. **c** In the serum, insulin T significantly decreased (**P < 0.005) in the 21, (***P < 0.0005) 28- and (†*P* < 0.01) and 42-d-old mice whereas insulin 2 significantly diminished in the (*P < 0.05) 21- and (**P < 0.005) and 28-d-old mice. **d** The rise in Insulin T is significant (##*P* < 0.002) from 42 to > 60d while the decrease in Insulin 2 is gradual from 14 to > 60 days. **e** Insulin T and Insulin 2 values and profiles are similar, both exhibited a sharp and very significant increment (a*P* < 0.00001) from 28 to 42 days and again (††*P* < 0.001) in the > 60-d-old mice. **f** and **g** representative western blots accompanied by corresponding histograms of the IR-β subunit in **f** ITf and **h** STf from 14 to > 60 d. **f** the changes in 110 kDa are significant by (††*P* < 0.001) 21, 28, 35, 42 and (##*P* < 0.002) > 60 days; the changes in 98 kDa are significant by (# < *P* < 0.02) 21, (†*P* < 0.01) 28 and (##*P* < 0.002) 35 days; the changes in 75 kDa are significant by (##*P* < 0.002) 21, (††*P* < 0.001) 28, 35, 42 and (a*P* < 0.0001) > 60 days; the changes in Total IR-β levels are significant in (††*P* < 0.001) 21, 28, 35, 42, and > 60 day-old mice. **g** the changes in 110 kDa are significant by (††*P* < 0.001) 21, 28, 35 and 42 days; the changes in 98 kDa are significant by (††*P* < 0.001) 21, 35, 42 and by (†*P* < 0.01) 28 and > 60 days. **h** and **i** representative western blots with corresponding histograms of IR-α subunit in **h** ITf and **i** STf from 14 to > 60 days after birth. **h** the changes in (b*P* < 0.00005) 135-, 90-, (†*P* < 0.03) 75-, (**P < 0.005) 50-, (###*P* < 0.0002) 36-, (k*P* < 0.00002) 31 kDa and (b*P* < 0.00005) Total IR-α levels are significant in the 28-day-old mice; the changes in (###*P* < 0.0002) 135-, 90-, (#*P* < 0.05) 75-, 31-, (b*P* < 0.00005) 36 kDa and (***P < 0.0005) Total IR-α levels are significant in the 42-day-old mice; the changes in (‡*P* < 0.03) 135-, (‡‡‡*P* < 0.0003) 90-, (##*P* < 0.002) 75-, (##*P* < 0.0002) 50-, (**P < 0.005) 36-, (‡*P* < 0.03) 31 kDa and (##*P* < 0.002) Total IR-α levels in the > 60-day-old mice are significant. **i** the changes in (††*P* < 0.001) 135-, (#*P* < *P* < 0.02) 90-and (#*P* < 0.02) 36 kDa are significant in the 21-day-old mice; the changes in (*P < 0.02) 75-, (‡*P* < 0.03) 50-, (##*P* < 0.002) 36 kDa and (**P < 0.005) Total IR-α levels are significant in the 28-day-old mice; the changes in (†*P* < 0.01) 75- and (***P < 0.0005) 36 kDa are significant in the 42-day-old mice; the changes in (*P < 0.05) 135-, (###*P* < 0.0002) 90-, (#*P* < 0.02) 75-, (‡*P* < 0.03) 36-, (†*P* < 0.01) 31 kDa and Total IR-α levels in the > 60-day-old mice

Glucose concentrations significantly and steadily rose from 14 to 42 days in male mice (Fig. 1a) before stabilising around 140 mg/dl in the > 60 day *postpartum* (adulthood) mice (Fig. 1a).

##### Interstitial tissue- enriched fractions

Glucose levels rose 2.5 times from 14 days to > 60 days, climbing around 2.75 μg/mg (Fig. 1b). The increase was significant by 21 days and again by 28 days (Fig. 1b).

##### Seminiferous tubule-enriched fractions

Glucose concentrations increased from 1.2 to 1.5 μg/mg from 14 to > 60 days when they reach maximal values which were merely half the ones in the > 60-day-old mice interstitium (Fig. 1b). Moreover, a significant increase occurred later, by 42 days in the tubules (Fig. 1b).

#### Insulin T and insulin 2

##### Serum

In sharp contrast to blood glucose, serum Insulin T (Total Insulin) and Insulin 2 concentrations were high in the 14 day-old male mice then fell from 21 to > 60 days (Fig. 1c). Serum Insulin T levels ranged between 0.88 mg/ml in the 14 day-old mice to 0.56 mg/ml in the > 60 day-old mice. Insulin T and Insulin 2 levels exhibited similar profiles though Insulin 2 values were slightly lower and ranged from 0.82 mg/ml in the 14 day-old mice to 0.42 mg/ml in the > 60 day-old (Fig. 1c). The drop in Insulin T was significant in the 21-, 28- and 42-day-old mice whereas the Insulin 2 drop was significant in the 21- and 28- day-old mice (Fig. 1c).

##### Interstitial tissue-enriched fractions

Insulin T levels were low around 2.2–2.4 μg/mg from 14 to 42 days after birth next, significantly rose in the > 60 day-old mice (Fig. 1d). The profile of Insulin 2 resemble that of Insulin T from 14 to 42 days except for a noticeable difference between Insulin T and Insulin 2 which had dropped to 1.9 μg/mg in the > 60 day-old mouse interstitial tissue fractions (Fig. 1d).

##### Seminiferous tubule-enriched fractions

By contrast to the interstitial tissue fractions, Insulin T and Insulin2 concentrations and profiles were similarly low in the tubules fractions in which they existed in traces from 14 to 28 days before sharply increasing ten folds in the 42-day-old mice and rising again in the > 60 day-old mice (Fig. 1e).

#### Insulin receptor beta subunit (IR-β)

##### Interstitial tissue-enriched fractions

Figure 1f shows a 98 kDa insulin receptor beta subunit (IR-β) immunoreactive band accompanied by an intense 110 kDa band in the 14-day-old mice. The 110 kDa levels significantly escalated in the 21-day-old mice then, significantly dropped by 28 and 35 days then, significantly rose again first by 42 days and again in the > 60 day-old mice to reach values slightly below the 14-day-old mouse values (Fig. 1f). The 98 kDa levels exhibited a similar profile from 14 to 35 days except that the values were low from 35 days to > 60 days (Fig. 1f). Traces of 75 kDa were detected in the 14-day-old mouse; 75 kDa levels significantly rose by 21 and 28 days and peaked by 42 days. Seventy five kDa was no longer detected in the > 60-day-old mice (Fig. 1f). Total IR-β levels significantly rose from 14 to 21 days then significantly decreased in the 21- and 35-day-old mice, rose in the 42-day-old mice and decreased again in adulthood (> 60 days) (Fig. 1f).

##### Seminiferous tubule-enriched fractions

The 110 kDa levels were highest in the 14-day-old mouse then, they significantly dropped in the 21-, 28- and 35-day-old mice however, the levels significantly rose in the 42- and again in the > 60 day-old mice to roughly half the 14-day-old mouse values (Fig. 1g). The 98 kDa levels significantly rose by 21 days then fell in the 28- and 35-day-old mice but peaked in the 42-day-old mice then significantly decreased again in the > 60 day-old mice (Fig. 1g). The 75-kDa levels significantly rose from 14 to 21 days but dropped by 35 days then significantly augmented by 42 days but were reduced to traces in the > 60-day-old mice (Fig. 1g). Traces of 50 kDa were present mainly in the 14-day-old mice (Fig. 1g). Total IR-β levels were elevated in the 14-day-old mice then, they significantly fell by 21, 28 and 35 days and decreased to their lowest in the > 60 day-old mice in which the values were roughly halved the 14-day-old mouse values (Fig. 1g).

#### Insulin receptor alpha subunit (IR-α)

##### Interstitial tissue-enriched fractions

The 135-, 90-, 75-, and 36 kDa IR-α immunoreactive bands were detected from 28 days onwards (Fig. 1h). The 135-, 90-, 75 kDa levels significantly and steadily increased from 28- to > 60 days (Fig. 1h). The 50 kDa IR-α levels nearly doubled from 14 to > 60 days (Fig. 1h). Thirty six kDa levels significantly increased from 28 to > 60 days whereas 31 kDa instead significantly dropped in the 42- and > 60 day-old mice in which it was reduced to traces (Fig. 1h). Total IR-α and 75 kDa profiles were similar in that both significantly increased from 28- to > 60 days when the values had tripled (Fig. 1h).

##### Seminiferous tubule-enriched fractions

The active 135 kDa form levels significantly dropped from 14 to 21 days then rose in the > 60-day-old mice (Fig. 1i). Likewise, 90 kDa levels first significantly decreased in the 21-day-old mice then significantly increased in the > 60-day-old mice (Fig. 1i). The 75 kDa levels significantly and steadily increased in the 28-, 42- and 60-day-old mice where the values were three times that in the 14-day-old mice (Fig. 1i). The 36- and 31 kDa levels dropped to traces levels from 42 to > 60 days (Fig. 1i).

#### Studies in *db/db* and *ob/ob* mice

##### Serum

**Fig. 2:**
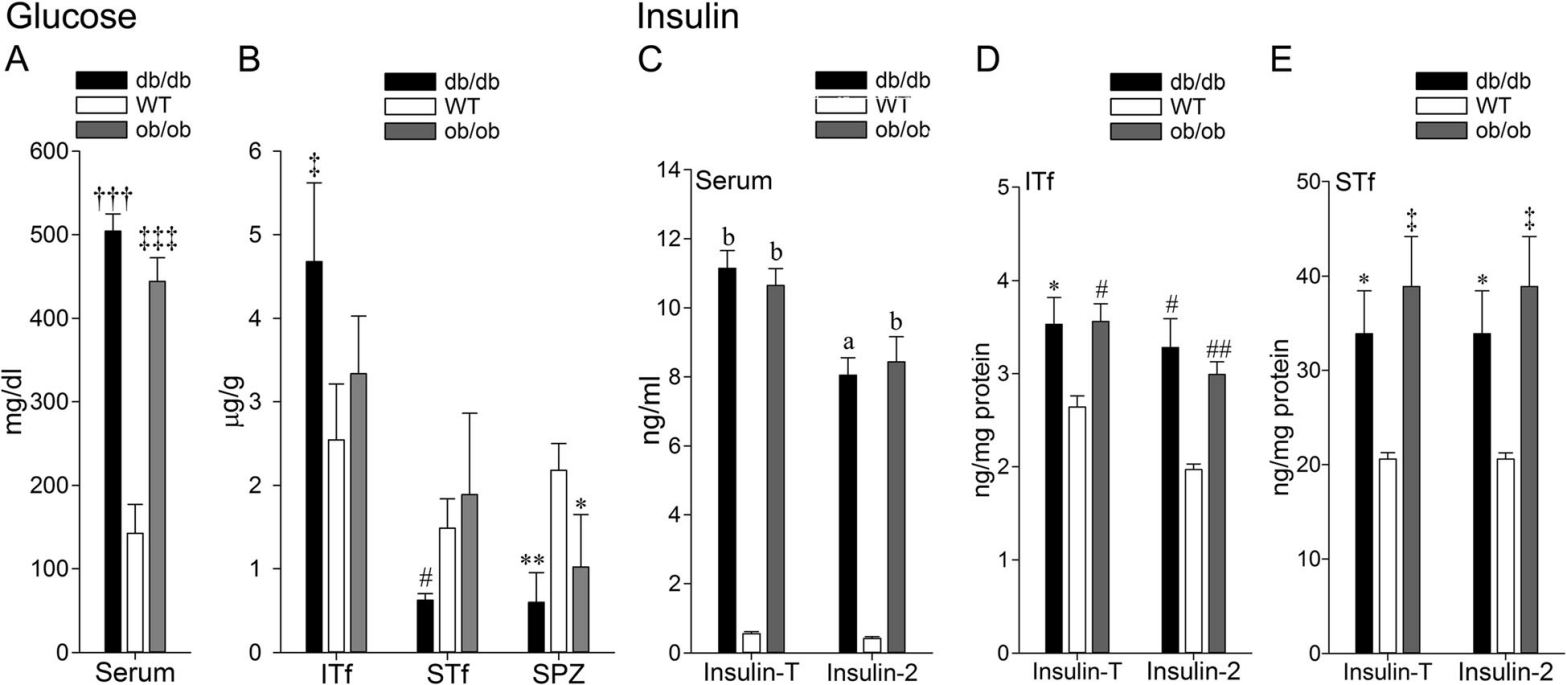
**a** and **b** show the glucose concentration in **a** the serum and **b** the interstitial tissue- (ITf) and seminiferous tubule-enriched fraction (STf) and in epididymal spermatozoa (SPZ) from *db/db*, *ob/ob* and wt mice. The values are the means ± SEM; *n* = 3 animals per age group. **a** The increase in circulating glucose concentration was very significant in (†††*P* < 0.0001) *db/db* and (‡‡‡*P* < 0.0003) *ob/ob* compared to the wt counterparts. **b** In *db/db*, glucose concentration significantly (‡*P* < 0.03) augmented in ITf but instead significantly dropped in (#*P* < 0.02) STf and (**P < 0.005) SPZ compared to the wt counterparts. The decrease in glucose was significant (*P < 0.05) in SPZ in *ob/ob* compared to the wt counterparts. **c-e** Insulin T and Insulin 2 in **c** serum, **d** ITf and **e** STf in *db/db*, *ob/ob* and wt. The values are the means ± SEM; *n* = 3 animals per age group. **c** Insulin T very significantly increased in both (b*P* < 0.00005) *db/db* and (b*P* < 0.00005) *ob/ob* sera compared to the wt counterparts. As well, Insulin 2 also very significantly escalated in (a*P* < 0.00001) *db/db* and (b*P* < 0.00005) *ob/ob* sera. **d** In ITf, Insulin T significantly augmented in (*P < 0.05) *db/db* and (#*P* < 0.02) *ob/ob* compared to the wt counterparts; similarly, Insulin2 significantly augmented in (#*P* < 0.02) *db/db* and (##*P* < 0.002) *ob/ob*. **e** As well, Insulin T significantly increased in STf in (*P < 0.05) *db/db* and (I*P* < 0.03) *ob/ob* compared to the wt

Blood glucose concentration tripled in *ob/ob* and more than tripled in *db/db* compared to the wild type (wt) counterparts (Fig. 2a).

##### Interstitial tissue-enriched fractions

Glucose levels increase was very significant in *db/db* mice but not in *ob/ob* mice when compared to the wt counterparts (Fig. 2b).

##### Seminiferous tubule-enriched fractions

Glucose significantly dropped in *db/db* but not in *ob/ob* compared to the wt counterparts (Fig. 2b).

##### Glucose in epididymal spermatozoa (SPZ)

Glucose concentration significantly in *db/db* and *ob/ob* mice (Fig. 2b).

#### Insulin T and insulin 2 levels

##### Serum

Insulin T concentration escalated roughly 22 folds in *db/db* and *ob/ob* compared to the wt counterparts (Fig. 2c). Likewise, Insulin 2 was majored about 16 folds in *db/db* and *ob/ob* respectively compared to wt counterparts (Fig. 2c).

##### Interstitial tissue-enriched fractions

As well, Insulin T augmented roughly 25% and Insulin 2 increased 40% in *db/db* and *ob/ob* compared to the wt counterparts (Fig. 2d).

##### Seminiferous tubule-enriched fractions

Similarly, Insulin 1 and Insulin 2 values doubled in *db/db* compared to wt counterparts (Fig. 2e). Surprisingly, the Insulin 1 and Insulin 2 increase was more significant in the *ob/ob* than in *db/db* mice compared to wt (Fig. 2e).

#### Insulin receptor beta subunit (IR-β)

##### Interstitial tissue-enriched fractions

**Fig. 3:**
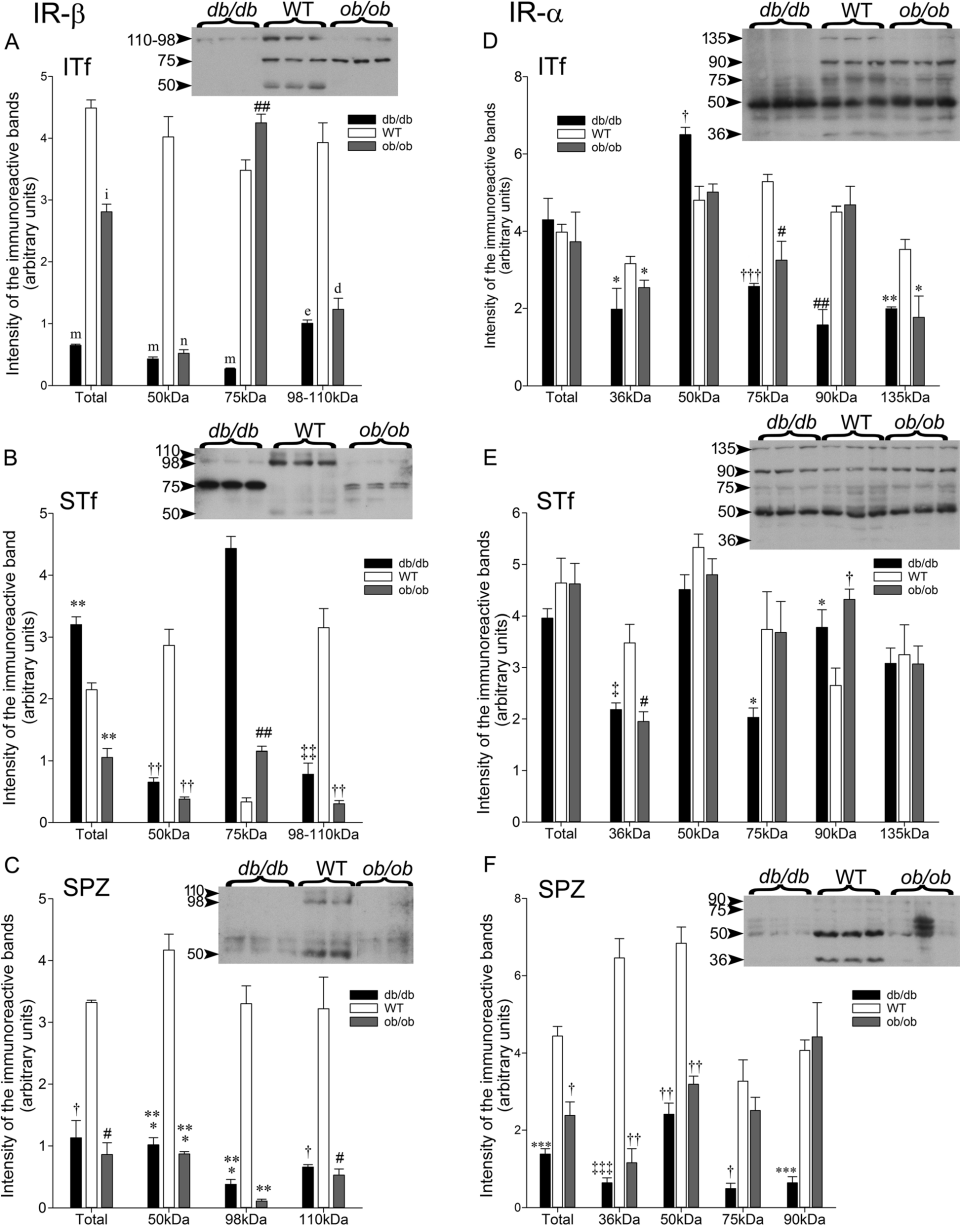
IR-β in **a** ITf, **b** STf and **c** SPZ in *db/db*, *ob/ob* and wt mice. The values are the means ± SEM; *n* = 3 animals per age group. **a** In ITf, the drop in 98-110 kDa band levels was significant in (c*P* < 0.000002) *db/db* and (d*P* < 0.000003) *ob/ob* mice compared to the wt counterparts. 75-kDa band levels fell significantly in *db/db* (m*P* < 0.0000001) but significantly increased (##*P* < 0.002) in *ob/ob* mice compared to the wt counterparts. Compared to the wt counterparts, 50 kDa band (m*P* < 0.0000001) and Total IR-β protein levels (m*P* < 0.0000001) significantly decreased in *db/db* mice; as well, in *ob/ob* mice, 50 kDa band (n*P* < 0.0000005) and Total IR-β protein levels (i*P* < 0.00003) significantly dropped. **b** In STf, 98-110 kDa band levels significant fell in *db/db* (‡‡*P* < 0.003) and *ob/ob* (††*P* < 0.001) compared to the wt counterparts. Contrarily, 75-kDa band levels significantly increased (b*P* < 0.00005) in *db/db* and *ob/ob* mice (##*P* < 0.002) compared to the wt counterparts. The 66 kDa band remained relatively unchanged in *db/db* but significantly augmented in *ob/ob* (*P < 0.05) mice in comparison to the wt counterparts. Fifty kDa significantly dropped in *db/db* (††*P* < 0.001) and *ob/ob* (††*P* < 0.001) compared to wt mice. Total IR-β protein levels significantly upsurged in (**P < 0.005) *db/db* but dropped in (††*P* < 0.001) *ob/ob* compared to the wt counterparts. **c** In SPZ, 98-110 kDa band significant fell in (*** *P* < 0.0005) *db/db* and (** *P* < 0.005) *ob/ob* compared to the wt counterparts. The decrease in 66 kDa band was not significant compared to the wt but the decrease in the 50 kDa band was significant (*** *P* < 0.0005) in *db/db* and *ob/ob* mice. Total IR-β protein levels significantly decreased in *db/db* (†*P* < 0.01) and *ob/ob* (#*P* < 0.02) compared to the wt counterparts. IR-α in **d** ITf, **e** STf and **e** SPZ. The values are the means ± SEM; *n* = 3 animals per age group. **d** In ITf, the decrease in 135 kDa band was significant in (**P < 0.005) *db/db* and (*P < 0.05) *ob/ob* and the drop in 90 kDa band was significant only in (##*P* < 0.002) *db/db* compared to the wt counterparts. The fall in 75 kDa band was significant in (†††*P* < 0.0001) *db/db* and (#*P* < 0.02) *ob/ob*. Fifty kDa band significantly increased in (†*P* < 0.01) *db/db* while 36 kDa band significantly decreased in both (*P < 0.05) *db/db* and *ob/ob* compared to wt counterparts. However, Total IR-α protein levels were similar in all groups. **e** In STf, 135 kDa band levels were not significantly different in *db/db* and *ob/ob* compared to the wt counterparts however, the increase in 90 kDa band was significant in (*P < 0.05) *db/db* and (†*P* < 0.01) *db/db*. The fall in 75 kDa was significant in (*P < 0.05) *db/db* compared to the wt counterparts. The decrease in 50 kDa bands levels was not significant whereas 36 kDa bands significantly dropped in (‡*P* < 0.03) *db/db* and (#*P* < 0.02) *ob/ob* compared to the wt counterparts. The decrease in Total IR-α protein levels were not significant in comparison to wt counterparts. **f** In SPZ, the decrease in 90 kDa band was significant only in (***P < 0.0005) *db/db* and the decrease in 75 kDa was significant in (†*P* < 0.01) *db/db* compared to the wt counterparts. The 50 kDa band significantly decreased (††*P* < 0.001) in *db/db* and *ob/ob* as well, 36 kDa significantly decreased in (‡‡‡*P* < 0.0003) *db/db* and (†*P* < 0.01) *ob/ob* compared to wt counterparts. Total IR-α levels were significantly lowered in (***P < 0.0005) *db/db* and (†*P* < 0.01) *ob/ob* in comparison to the wt

98–110 and 50 kDa IR-β bands significantly dropped to trace levels in *db/db* and *ob/ob* (Fig. 3a). The 75 kDa levels significantly fell in *db/db* whereas they augmented in *ob/ob* compared to the wt counterparts (Fig. 3a). Total IR-β were significantly reduced to traces levels in *db/db* and halved in *ob/ob* mice compared to the wt (Fig. 3a).

##### Seminiferous tubule-enriched fractions

IR-β values in the seminiferous tubule (Fig. 3b) significantly differed from the ones measured in the interstitial tissue fractions (Fig. 3a). Specifically, 98–110-, and 50 kDa very significantly fell *db/db* and *ob/ob* compared to the wt counterparts (Fig. 3b). By contrast, 75 kDa levels significantly increased in both *db/db* and *ob/ob* compared to the wt counterparts (Fig. 3b). Higher 66 kDa band levels were measured in the *ob/ob* mice (Fig. 3b). Total IR-β levels significantly increased in *db/db* but decreased *ob/ob* mice compared to the wt (Fig. 3b).

##### Epididymal spermatozoa (SPZ)

98–110- and 50 kDa and Total IR-β levels all significantly dropped in *db/db* and *ob/ob* compared to the wt counterparts (Fig. 3c). The 75 kDa IR-β immunoreactive band was not detected (Fig. 3c).

#### Insulin receptor alpha subunit (IR-α)

##### Interstitial tissue-enriched fractions

In *db/db*, 135-, 90-, 75- and 36 kDa IR-α levels significantly dropped whereas 50 kDa significantly increased compared to the wt counterparts while Total IR-α levels were not significantly different from wt (Fig. 3d). In *ob/ob*, 135-, 75- and 36 kDa levels significantly decreased albeit, 90- and 50 kDa, and Total IR-α levels exhibited little changes compared to the wt (Fig. 3d).

##### Seminiferous tubule-enriched fractions

The IR-α protein content showed strong differences in the seminiferous tubule and interstitial tissue fractions. Specifically, in *db/db*, mice 90 kDa significantly upsurged while 75- and 36 kDa levels significantly dropped and neither 135 kDa nor Total IR-α level significantly differed from the wt counterparts (Fig. 3e). In *ob/ob*, mice 90 kDa significantly raised whereas 36 kDa fell but 135-, 75- and 50 kDa and Total IR-α levels showed no significant difference with the wt (Fig. 3e).

##### Epididymal spermatozoa (SPZ)

The 135 kDa IR-α immunoreactive band was not detected in *db/db*, *ob/ob* and wt mouse epididymal spermatozoa (Fig. 3f). The 90-, 75-, 50-and 36 kDa and Total IR-α levels all very significantly plummeted in *db/db* compared to the wt counterpart mice (Fig. 3f). In *ob/ob* mice, 50 kDa, 36 kDa and Total IR-α levels significantly plunged while 90- and 75 kDa did not differ significantly from the wt (Fig. 3f).

### Anterior pituitary

#### IR-β subunit

**Fig. 4:**
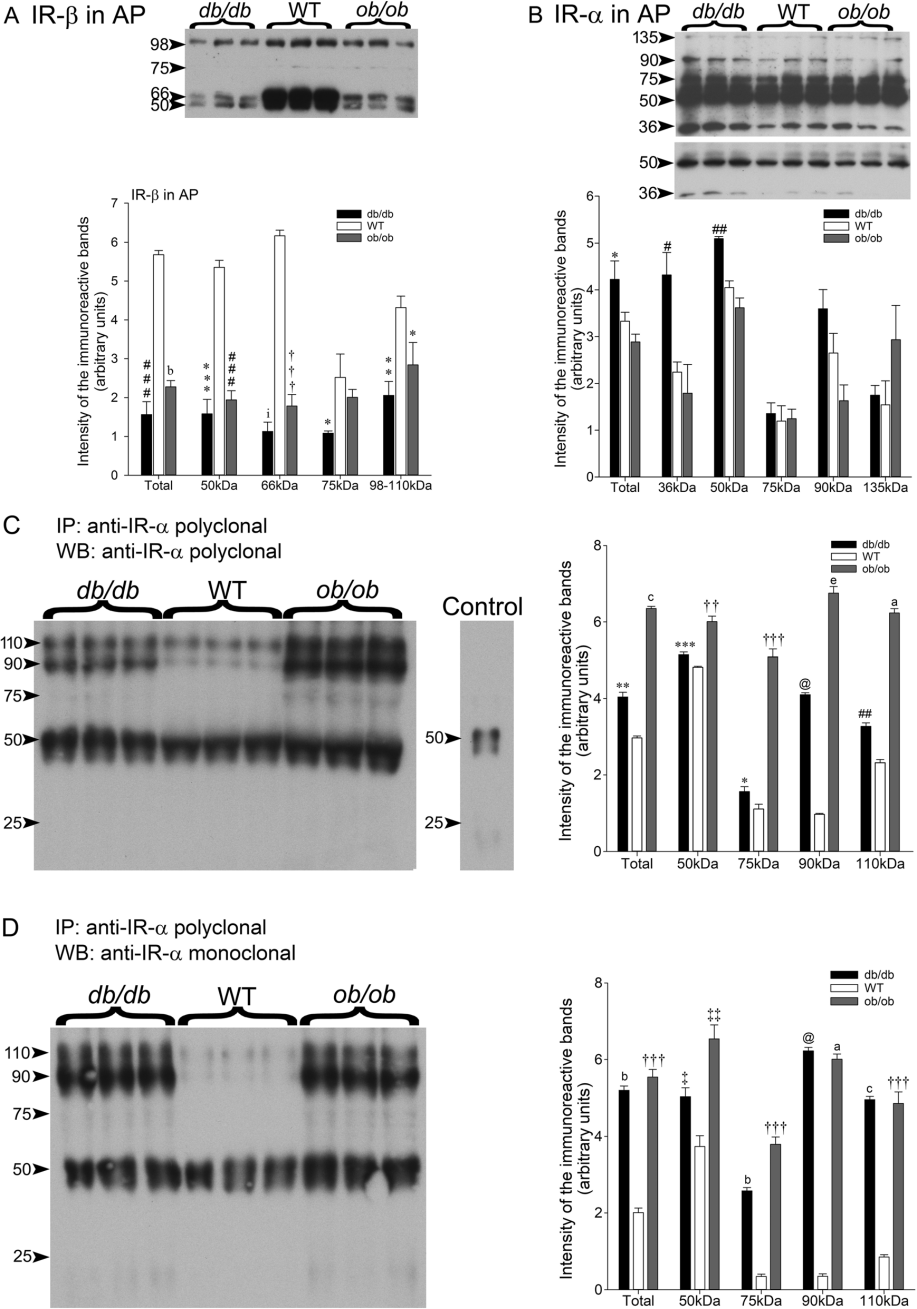
**a** Insulin receptor β-subunit levels in anterior pituitaries of leptin receptor-deficient (*db/db*) and leptin-deficient (*ob/ob*) and wt male mice. Thirty microgram of total protein samples were loaded per well. After electrophoretic migration, proteins were electrotransferred onto nitrocellulose membranes and probed with anti-IR-β subunit. Bands were scanned and their intensity quantified. The figure shows a representative western blot. The values are the mean ± SEM of 3 different animals in each experimental group. Total IR-β subunit levels were significantly lowered in (###*P* < 0.0002) *db/db* and (b*P* < 0.00005) *ob/ob* compared to wt counterparts. The full-length IR-β subunit levels decreased in (**P < 0.005) *db/db* and (* < *P*0.05) *ob/ob* mice compared to wt. The fragmentation increased in *db/db* and *ob/ob* mice by comparison to the wt: 50 kDa levels: (***P < 0.0005) *db/db* (###*P* < 0.0002) *ob/ob*; 66 kDa levels: (i*P* < 0.00003) *db/db* (†††*P* < 0.0001) *ob/ob*; 75 kDa levels: (* < P0.05) *db/db*. **b** Insulin receptor α-subunit expression in the leptin receptor-deficient (*db/db*) and leptin-deficient (*ob/ob*) and wt mice. Thirty μg of total protein samples were loaded per well. After electrophoretic migration, proteins were electrotransferred onto nitrocellulose membranes and probed with anti-IR-α subunit. Bands were scanned and their intensity quantified. The figure shows a representative western blot. The values are the mean ± SEM of 3 different animals in each experimental group. Total IR-α subunit levels were significantly higher in (* *P* > 0.05) *db/db* mice compared to the wt. The values did not significantly differ in *ob/ob* mice from the wt counterparts. The full-length IR-α subunit (135 kDa) levels were not significantly different in the three experimental groups. Only the (#*P* > 0.02) 36 kDa and (##*P* < 0.002) 50 kDa IR-α subunit fragments levels significantly increased in *db/db* mice compared to wt. **c** and **d** Soluble (*s*)IR-α levels in the serum in *db/db*, *ob/ob* and wt mice. Results obtained in **c** using an anti-IR-α polyclonal antibody for both, immunoprecipitation and western blotting and in **d** using anti-IR-α polyclonal antibody for immunoprecipitation then, using an anti-IR-α monoclonal antibody for western blotting. **c** In the serum of *db/db* mice, the rise in (##*P* < 0.002) 110-, (@*P* < 0.000001) 90-, (*P < 0.05) 75-, (***P < 0.0005) 50- and (**P < 0.005) Total *s*IR-α levels is significant compared to the wt counterparts. As well, the upsurge in 110- (a*P* < 0.00001) 110-, (e*P* < 0.000005) 90-, (†††*P* < 0.0001) 75-, (††*P* < 0.001) 50- and (c*P* < 0.000002) Total *s*IR-α in the *ob/ob* mice is significant compared to the wt counterparts. The control done on PBS shows an intense band around 50 kDa and a faint one around 21 kDa. **d** The (c*P* < 0.000002) 110-, (@*P* < 0.000001) 90-, (b*P* < 0.00005) 75-, (‡*P* < 0.03) 50- and (b*P* < 0.00005) Total *s*IR-α increase in the *db/db* mice compared to the wt counterparts. The (†††*P* < 0.0001) 110-, (a*P* < 0.00001) 90-, (†††*P* < 0.0001) 75-, (‡‡‡*P* < 0.003) 50- and (†††*P* < 0.0001) Total *s*IR-α levels increase in the *ob/ob* mice compared to the wt counterparts

The full length, total IR-β and IR-β fragments levels all significantly dropped in the *db/db* and *ob/ob* mouse anterior pituitaries compared to wt counterparts (Fig. 4a).

#### IR-α subunit

The leptin receptor and leptin deficiency had different effects on IR-α and IR-β subunit. IR-α was unchanged in leptin-deficient *ob/ob* mice compared to the wt counterparts (Fig. 4b). However, in the *db/db* mice Total, and 36- and 50 kDa IR-α fragment levels significantly increased compared to the wt despite unchanged 135 kDa IR-α full-length levels (Fig. 4b).

### Soluble insulin receptor alpha (sIR-α) in the serum of *db/db*, *ob/ob* and wt mice

Two different antibodies were used to measure Soluble insulin receptor alpha (sIR-α) levels in the *db/db* and *ob/ob* mouse serum by ELISA: a polyclonal (Fig. 4c) and a monoclonal (Fig. 4d) IR-α antibody. In the first technical approach, the anti-IR-α polyclonal antibody was used for both, immunoprecipitation and western blotting while PBS was used for control in the serum (Fig. 4c). In the second approach, the IR-α polyclonal antibody was used for immunoprecipitation whereas the IR-α monoclonal antibody was used in western blotting (Fig. 4d). Immunoprecipitation of serum proteins with a polyclonal anti-IR-α followed by immunoblotting with the same antibody detected a 110-, 90- and 50 kDa IR-α immunoreactive bands (Fig. 4c). Because the 50 kDa band coincides with the molecular mass of the IgG heavy chain (Fig. 4 c, left panel, control), the experiment was repeated with a monoclonal anti-IR-α in immunoblotting analyses (Fig. 4d). The results obtained with the monoclonal antibody were essentially similar to the ones obtained with the polyclonal anti-IR-α. Aside from differences in the sIR-α levels measured by ELISA, the two technical approaches revealed very significantly augmented 110-, 90-, 75-, 50 kDa and Total sIR-α levels in the *db/db* and *ob/ob* mouse serum compared to the wt counterparts (Fig. 4c-d).

Table 1 provides an overview account of glucose, Insulin T, Insulin 2, IR-β, IR-α in the serum, the interstitial tissue (ITf) and seminiferous tubule fractions (STf) and epididymal spermatozoa (SPZ) in *db/db* and *ob/ob* mice compared to the wt counterparts. In *db/db* mice, increased glucose in circulating blood was accompanied by an increase in Insulin T, Insulin 2 and Total soluble (*s*) IR-α levels in the serum though the values of glucose, Insulin T and Insulin 2 measured in ITf, STf and epididymal SPZ strongly differed from the values measured in serum. Glucose, Insulin T and Insulin 2 increased concomitantly in ITf and serum however, the sugar dropped in the STf and epididymal SPZ while Insulin T and Insulin 2 increased in STf in *db/db* mice compared to the wt. Moreover, there is a decrease in both IR-α and β subunits in ITf whereas only IR-β decreased in the STf and SPZ. In the *ob/ob* mice, glucose was higher in serum whereas it changed little in ITf and STf and dropped in SPZ. Contrarily, Insulin T and Insulin 2 increased in serum, ITf and STf. In these animals, IR α and β subunits were lowered in ITf whereas only the β subunit decreased in the STf and SPZ.

**Table 1:** An overview of the changes in glucose, Insulin T, Insulin 2, IR-β, IR-α in the serum, the interstitial tissue (ITf), seminiferous tubule fractions (STf), epididymal spermatozoa (SPZ) and anterior pituitary (AP) in *db/db* and *ob/ob* mice compared to the wt counterparts

	*db/db*					*ob/ob*				
	serum	ITf	STf	SPZ	AP	serum	ITf	STf	SPZ	AP
Glucose	**↑**	**↑**	**↓**	**↓**		**↑**	**≅**	**≅**	**↓**	
Insulin T	**↑**	**↑**	**↑**			**↑**	**↑**	**↑**		
Insulin 2	**↑**	**↑**	**↑**			**↑**	**↑**	**↑**		
IR-β 98-110 kDa		**↓**	**↓**	**↓**	**↓**		**↓**	**↓**	**↓**	**↓**
IR-α 135 kDa		**↓**	**≅**	nd	**≅**		**↓**	**≅**	nd	≅
*s*IR-α 110 kDa	**↑**					**↑**				

*nd* Not detected

## Discussion

### Postnatal development studies

The present study provides the first weekly assessments of glucose, Insulin T, Insulin 2, and of the IR-β and IR-α protein content in interstitial tissue- and seminiferous tubule-enriched fractions during the mouse postnatal development.

#### Glucose

The results reveal that glucose concentration is low in the 14-day-old mouse interstitial tissue and seminiferous tubules fractions. In the tubules, spermatocytes and spermatids supply glucose, the major source of energy [21]. The low quantities of glucose in the 14-day-old mouse tubules likely reflect the low numbers of these germ cells. Glucose concentrations increased in the blood, the interstitial tissue and seminiferous tubules during development. However, the increase proceeded at half the speed in tubules compared to the interstitium and maximal concentrations were 60% superior in the interstitium than in tubules in the 60-day-old mouse indicating distinct glucose metabolisms in these two cellular compartments of the testis.

#### Insulin

Serum Insulin T and Insulin 2 exhibited similar profiles, both dropped as glucose increased. However, in contrast to glucose, Insulin T and Insulin2 levels were ten folds higher in tubules than in the interstitium in the 60-day-old mouse. In the tubules, high Insulin T and Insulin 2 accompanying low glucose concentrations indicates that Insulin responds and is regulated by glucose. By contrast Insulin T rose little and Insulin 2 even decreased while glucose concentration was 60% more elevated in the > 60-day-old mouse interstitial tissue than in tubules indicating that glucose levels are not regulated by insulin in the interstitium.

The Insulin T kit used in the present study measures total Insulin that is to say, Insulin 1 and Insulin 2 combined. The mouse seminiferous tubules likely contain only Insulin 2 since Insulin T and Insulin 2 exhibited similar profiles and virtually identical values indicating that total insulin (referred here as Insulin T) and Insulin 2 are the same. By contrast, in the interstitium, the distinct Insulin T and Insulin 2 profiles and values measured attest to the presence of both in the adult mice because with Insulin 2 decreasing, the rise in Insulin T can only resulted from an increase in Insulin 1 which the commercial kit used had not measured. Therefore, on this issue, our results do not support that mouse testis “*contains only detectable levels of ins2 and lack any detectable expression of ins1*” [51]. This discrepancy likely stems from the fact that here, we assessed insulin variations in interstitial tissue and tubules fractions in contrast to Schoeller et al., 2014 study in which the hormone was measured in whole testis extracts. Moreover, the concurrent Insulin T and glucose increase we measured in the interstitium indicates that insulin levels do not respond to glucose in this cellular compartment of the mouse testis in vivo. Our view is in line with the conclusion that insulin secretion is not glucose regulated in human cultured Leydig cells [52]. Evidence shows that insulin which has been localised in Leydig cells [31] is secreted in the circulation [52]. Furthermore, our results agree with the report of an insulin increase in testicular interstitial fluid from 21 to 70 days in rats [53].

Our results show that Insulin T augmented in the mouse interstitial tissue and tubules fractions during development and reveal that the increase came from Insulin 1 in the interstitium and from Insulin 2 in the tubules. It is worth noting that the hormone increase took place between 28 and 42 days after birth which in the mouse concurs with the switch from puberty to adulthood.

Cultured Sertoli cells remain viable in the absence of glucose since the sugar is not their principal energy source [3]. In the seminiferous tubules, glucose is contributed principally by spermatocytes and spermatids [21]. Therefore, the insulin upsurge in tubules may reflect the glucose-contributing spermatocytes and spermatids arrival between 28 and 42 days. The hormone and insulin growth factors IGF-I and IGF-II were shown to promote spermatogonial differentiation into primary spermatocytes by binding to the IGF-I receptor [54]. Our finding of an insulin build-up in adult mouse tubules is consistent with the reports of higher concentrations of insulin in the seminal plasma than in serum in men [55, 56] and rat [53]. The blood-testis barrier is not an efficient obstacle to insulin’s entry in tubule as evidenced by our observation of its accruement in the adult mouse tubules together with the report of the hormone being concentrated in the semen [55]. Our finding adds support to the notion that “*the hormone appears to freely cross the barrier*” [55]. However, our data disagree with the statement that “*insulin... cannot pass through the blood-testis barrier*” [51]. This discrepancy between our results and the *Schoeller’s* et al. 2012 conclusion [51] may stem from differences in the technical approaches used in the two studies. These authors wrote “*Since the blood-testis barrier in mice forms at postnatal day (p)12, we injected p7, p9, and p12 testes with FITC insulin, and this experiment showed an increasing restriction to the interstitium as the barrier forms (Fig. 5D)*” [51]. The establishment of the blood-testis barrier takes place during the colonisation of the seminiferous tubules by the zygotene spermatocytes by 21 days after birth in rodents [57]. This means that injection with FITC insulin in p7, p9, and p12 mouse testes as described in Schoeller’s study [51] took place before the establishment of a competent blood-testis barrier and to the completion of puberty.

### Insulin receptor alpha (IR-α) and beta (IR-β) subunits

Our finding of IR-β in mouse interstitium and seminiferous tubule fractions supports and extends earlier reports of IR-β immunoreactivity in mouse whole testis extracts [58], rat and mouse Leydig cells [53, 59] and cultured immature rat Sertoli cells [60]. Our finding that the active 110 kDa IR-β form peaks in the 28-day-old mouse interstitial tissue fractions may reflect the growing Leydig cell numbers acquiring the insulin-sensitive phenotype around puberty. This view is consistent with the punctual increase in the specific insulin binding reported between 21 and 40 days in purified rat Leydig cells [53]. The 75 kDa IR-β fragment levels decline in the > 60 day-old-mouse interstitium and tubules indicates the presence of small amounts of degradation products of the insulin receptor. In tubules, the maximal 110 kDa IR-β levels recorded in the 14-day-old mice may be related to the arrivals of primary spermatocytes as they acquired the insulin-sensitive phenotype by 17 days *postpartum*. The present study provides the first measurements of the IR-α subunit variation in the two individual cellular compartments of the testis. The active 135 kDa IR-α form levels are high in the > 60 day-old adult mouse interstitial tissue and tubule fractions. One hundred thirty five kDa IR-α is not detected in epididymal spermatozoa. With regards to 98- and 110 kDa IR-β, in the 60-day-old mice, 98 kDa IR-β levels are low in the interstitium while being elevated in tubules whereas the opposite is true for the 110 kDa IR-β form. However, both the interstitial tissue and tubules exhibit only trace levels of 98–110, Total and 75 kDa IR-β fragments by 35 days. It is worth noting that this drop is concurrent with the peak in apoptosis that follows the completion of the first wave of the seminiferous epithelium in mouse.

### Studies in diabetic *db/db* and obese *ob/ob* mice

#### Glucose

The results confirm our earlier report of significantly increased glucose in the *db/db* and *ob/ob* mouse serum [45]. The data extend these findings and show that glucose concentrations doubled in the interstitial tissue but were halved in tubules in *db/db* mice. Significantly, glucose dropped only in the *db/db* mouse tubules even though both the *db/db* and the *ob/ob* mouse interstitium and tubules fractions exhibited insulin imbalances. This evidences a different response of the interstitial tissue and tubules to glucose and/or insulin signalling. Glucose uptake has been shown to drop in testes in which germ cells are absent for instance, in newborn testis [18] or following assaults [20]. In the seminiferous tubules, glucose, the major source of energy has been shown to be supplied by spermatocytes and spermatids [21]. It follows, that the number of these cells will influence glucose levels in the tubules. In this context, the significant drop in glucose concentration in the *db/db* mouse tubules could reflect the significant lost of glucose-containing spermatocytes and spermatids through increased apoptosis resulting from the *db* mutation-induced impairment of meiosis, a pathological feature typical of the *db/db* but not seen in the *ob/ob* mice [45]. The ~ 60–80% glucose concentrations decline in the *db/db* and *ob/ob* mouse epididymal spermatozoa entails a diminution in gametes fertility in both mouse models since the sugar stimulates capacitation and acrosome reaction [25, 61].

#### Insulin

Our results show that the increase in Insulin T and Insulin 2 is less in the testis than in serum suggesting a different local control of the hormone. Earlier, we reported that free and esterified cholesterol were significantly elevated in the blood but decreased in the interstitial tissue in *db/db* and *ob/ob* mice and that esterified cholesterol was reduced to traces in the *db/db* mouse tubules fractions [45]. The *db/db* and *ob/ob* mice have low testosterone levels [15]. Furthermore, we have shown that the *db/db* and *ob/ob* mice used in the present study had very significantly lowered testosterone levels [45]. Our earlier report on testosterone, combined with the increased Insulin T and Insulin 2 measured here in the presence of elevated glucose in the interstitium indicate that insulin overexpression reflects a deregulation of both, the carbohydrate and lipid metabolisms in Leydig cells and macrophages that impacts their function. The high insulin and glucose concentrations in the *db/db* mouse interstitial tissue fractions indicate that the hormone levels are not regulated by glucose in this cellular compartment of the testis and reveals a resistance to insulin in these mouse Leydig cells. On the one hand, the deleterious effect of high insulin on Leydig cells is direct since insulin was reported to stimulate dosage-sensitive sex reversal adrenal hypoplasia critical region on chromosome X gene 1 (DAX-1) expression which in turn inhibits testosterone synthesis independently of luteinising hormone (LH) [62]. On the other hand, Leydig cells lack glucose sensing mechanisms to process proinsulin into biologically active insulin necessary for glucose regulation [52]. Moreover, here, *db/db* and *ob/ob* mice exhibited imbalances insulin receptor levels.

By contrast, our finding of decreased glucose concentrations under elevated Insulin T concentrations in the *db/db* mouse seminiferous tubules indicates a response to Insulin 2 since Insulin T = Insulin 2 in the tubules. Distinct functions have been assigned to *ins1* and *ins2* genes [39, 40].

### Insulin receptor α and β subunits

The initial interaction of insulin with the target cells involves the binding of the hormone to the extracellular α-subunit of the IR which triggers the autophosphorylation of the β-chain cytoplasmic regions and activates the constitutive tyrosine kinase activity of the β-subunit. This results in the phosphorylation of tyrosines in different regulatory proteins and the downstream activation of signalling cascades that regulate the metabolism of insulin within cells [63]. Furthermore, the binding of insulin to the IR-α subunit triggers the endocytosis of the hormone-bound receptor for recycling or degradation [64, 65]. Following endocytosis of the insulin receptor, proteolytic enzymes breakdown the IR-β chain into fragments of lower molecular masses (~ 75-, 61- and 52 kDa) recovered in the lysosomal and /or cytosolic fractions [66, 67]. Little is known about intracellular processing of the endocytosed α-subunit. In addition to the IR intracellular processing, the normal shedding of the receptor releases a soluble ectodomain comprising the two α-subunits bound to a portion of the β-subunit extracellular regions [68, 69].

Our finding of significantly reduced 110 kDa IR-β and 135 kDa IR-α subunit protein content in the *db/db* and *ob/ob* mouse interstitial tissue fractions indicates a loss of active receptor sites in the cells’ membrane that causes Leydig cells and other cells to bind less insulin and not to respond to the hormone. The *ob/ob* mouse hepatocyte “*bind only 20 to 25 % as much insulin per mg of protein as those of their thin litter mates*” [70]. Insulin increased in the *db/db* and *ob/ob* mouse interstitial tissue fractions. The loss of IR may be due to an increased endocytosis of the receptor under high insulin levels. The presence of several IR-α and IR-β immunoreactive bands in *db/db* and *ob/ob* interstitial tissue fraction lysates is aligned with this view. It has been shown that under chronic insulin stimulation, there is a decrease in insulin binding sites on the membrane due to an increased degradation of the endocytosed IRs [71]. Moreover, an exacerbated IR breaking down has been reported in tissues and cells under hyperinsulinaemic conditions such as obesity, metabolic syndrome and type 2 diabetes [67, 70–73]. Our results also show an upsurge in serum *s*IR-α in *db/db* and *ob/ob* mice. Shedding of IR increases under hypertensive, hyperinsulinaemic and hyperglycaemic conditions [68, 69, 74, 75]. The excess in Insulin T and Insulin 2 in the *db/db* and *ob/ob* mouse interstitial tissue fractions may further decrease IR levels by exacerbating shedding and degradation of IR. Local dysfunctions in the control of the metabolism of insulin resulting from the *db* and *ob* mutation-induced downregulation of IR-α and IR-β subunits in the interstitium could explain in part the drop in testosterone typical of the *db/db* and *ob/ob* mice [45].

Our results show that 110 kDa IR-β subunit levels decreased while 135 kDa IR-α subunit levels remained unchanged notwithstanding significantly elevated insulin in the *db/db* and *ob/ob* mouse tubule fractions. Thus, insulin binding sites are not affected in this cellular compartment of the testis but still, the reduced β-subunits to be activated are insufficient to stimulate downstream effectors.

Spermatozoa secrete and express insulin [32] and IR-β [61]. This study gives the first account of IR-α and IR-β levels in epididymal spermatozoa and show that the *db* and *ob* mutations decreased 110 kDa IR-β in gametes. However, the 90 kDa IR-α protein content decreased in the *db/db* but not in *ob/ob* mice.

Evidence shows that leptin directly impacts the anterior pituitary hormone secretion [6, 76, 77]. Impairment of spermatogenesis under hyperinsulinaemic conditions has been attributed in part to decreased luteinizing hormone [58]. Insulin receptor levels are higher in the anterior than in the intermediate and posterior pituitary lobes of the pituitary [5]. Deleting the insulin receptor encoding gene deregulates the anterior pituitary hormone secretion [5]. In the present study, the *db/db* and *ob/ob* mouse anterior pituitaries present aberrations in IRα and β subunit levels. Specifically, the fall in Total and full-length IR-β subunit levels under the hyperinsulinaemic conditions resulting from the *db/db* and *ob/ob* mutations suggests enhanced degradation of the subunit which can lead to defects in the downstream signalling in the anterior pituitary. High insulin levels have been shown to exacerbate insulin receptor endocytosis [71]. Following endocytosis, the IR-β chain is cleaved into ~ 75-, 61- and 52 kDa fragments [66, 67].

The *db/db* and *ob/ob* mutations induce hyperglycaemia and hyperinsulinaemia but each mutation produces different effects on IR-α. Neither the levels of the full-length IR-α subunit nor the degradation of IR-α were affected in the *ob/ob* mice though the Total IR-α protein content significantly augmented in the *db/db* mouse anterior pituitary. The IR-α fragmentation increase accompanying unchanged active 135 kDa full-length IR-α levels suggests an exacerbated degradation of the IR-α subunit in the anterior pituitary resulting from the *db/db* mutation.

## Conclusion

Our development studies show that insulin is not glucose regulated in the interstitium whereas the hormone regulates glucose concentrations in tubules. The mouse seminiferous tubules contain Insulin 2 whereas the interstitium contains Insulin T and Insulin 2. The high levels of IR-α fragments in *db/db* and *ob/ob* sera indicates increased shedding of the receptor under hyperinsulinaemic conditions. In the interstitium, the down regulation of IR-α and β subunits induced by the *db* and *ob* mutations is indicative of a loss of active receptor sites capable to alter the testicular cells insulin binding and response to the hormone. In the tubules and anterior pituitary, insulin binding sites are less affected but the downregulation of the β-subunits leads to a drop in the number of β-subunits susceptible to be activated by insulin thus, affecting the stimulation of IR downstream effectors.

Our results agree with findings in other tissues and further show that chronically elevated insulin levels caused the degradation of IR subunits that follow distinct pathways in the two cellular compartment of the testis. Moreover, we present evidence of strong differences in the response to diabetes-induced aberrations in the protein content and fragmentation of insulin receptors and in the consequences on insulin signalling in the interstitial tissue and seminiferous tubules between the leptin receptor-deficient *db/db* and leptin-deficient *ob/ob* mouse models.

## Data Availability

All data generated or analysed during this study are included in this published article.

## Abbreviations

bpV (phen): Potassium bisperoxo (1, 10- phenanthroline) oxovanadate (V)
Cx: Connexin
Cxs: Connexins
EGTA: Ethylene glycol-bis(β-aminoethyl ether)-N,N,N′,N′-tetraacetic acid
Ig: Immunoglogulin
ITf: Interstitial tissue-enriched fraction
IR: Insulin receptor
SDS-PAGE: Sodium dodecyl sulfate polyacrylamide gel electrophoresis
STf: Seminiferous tubule-enriched fractions
PBS: Phosphate buffered saline
PMSF: Phenylmethylsulfonyl fluoride
RT: Room temperature
wt: Wild type
